# Community Health and Socioeconomic Issues Surrounding Concentrated Animal Feeding Operations

**DOI:** 10.1289/ehp.8836

**Published:** 2006-11-14

**Authors:** Kelley J. Donham, Steven Wing, David Osterberg, Jan L. Flora, Carol Hodne, Kendall M. Thu, Peter S. Thorne

**Affiliations:** 1 College of Public Health, The University of Iowa, Iowa City, Iowa, USA; 2 Department of Epidemiology, University of North Carolina, Chapel Hill, North Carolina, USA; 3 Department of Sociology, Iowa State University, Ames, Iowa, USA; 4 Department of Anthropology, Northern Illinois University, DeKalb, Illinois, USA

**Keywords:** animal confinements, environmental impact, livestock, mental health, odor, poultry, right-to-farm legislation, swine

## Abstract

A consensus of the Workgroup on Community and Socioeconomic Issues was that improving and sustaining healthy rural communities depends on integrating socioeconomic development and environmental protection. The workgroup agreed that the World Health Organization’s definition of health, “a state of complete physical, mental and social well-being and not merely the absence of disease or infirmity,” applies to rural communities. These principles are embodied in the following main points agreed upon by this workgroup. Healthy rural communities ensure *a*) the physical and mental health of individuals, *b*) financial security for individuals and the greater community, *c*) social well-being, *d* ) social and environmental justice, and *e*) political equity and access. This workgroup evaluated impacts of the proliferation of concentrated animal feeding operations (CAFOs) on sustaining the health of rural communities. Recommended policy changes include a more stringent process for issuing permits for CAFOs, considering bonding for manure storage basins, limiting animal density per watershed, enhancing local control, and mandating environmental impact statements.

## Background and Recent Developments

### The agricultural community in areas of large-scale livestock production

The rural and agricultural community has changed dramatically over the past half century. The trends include an overall reduction in the number of farms, an increase in size of the farms, and economic concentration in the industries that supply inputs and purchase commodities from farms. The structure of the pork industry has also changed dramatically during the past three decades. The number of hog producers in the United States was more than 1 million in the 1960s but fell to about 67,000 by 2005 [U.S. Department of Agriculture (USDA) 2005]. Although the total inventory of hogs has changed little over the years, the structural shift toward concentration has been dramatic with the 110 largest hog operations in the country, each of which has over 50,000 hogs, now constituting 55% of the total national inventory (USDA 2005). The swine industry includes the following types of producers: small independent “niche” operators who often market organic pork to local markets, traditional independent operators, and large family or unaffiliated corporations. Former independent operators are increasingly raising livestock on contract for larger corporations. According to the U.S. Government Accountability Office, in 1999 contract production constituted more than 60% of total hog output and 35% of the cattle market (U.S. Government Accountability Office 2005), while poultry is produced almost entirely via contracts. Corporate producers or incorporated family-based operations employ from a few individuals to several hundred. Most often upper management and many of the workers in such operations do not come from or live in the vicinity of concentrated animal feeding operations (CAFOs).

The community of people living in the region of large-scale livestock production consists of residents of small family farms (that may or may not produce pork), workers at the production facilities, rural nonfarm residents, and the residents of neighboring towns. The challenges CAFOs place on neighbors were extensively reviewed in 1996 (Thu 1996) and again in a 2002 report accompanied by a number of consensus recommendations for the future of the hog industry in Iowa (Iowa State University and University of Iowa 2002). A number of additional scientific reviews and symposia summaries have been issued (Centers for Disease Control and Prevention 1998; Cole et al. 2000; Donham 2000; National Academy of Sciences 2002; Schiffman et al. 2000; Thu 2002).

### Economic health

Economic concentration of agricultural operations tends to remove a higher percentage of money from rural communities than when the industry is dominated by smaller farm operations, which tend to circulate money within the community. Goldschmidt (1978) documented this as early as 1946 in California, one of the first states where industrialized agriculture developed. Specifically, he compared two agricultural communities, one dominated by larger industrialized farms with absentee ownership and a high percentage of hired farm labor, and the other community was dominated by smaller owner-operated farms. The latter community was found to have a richer civic and social fabric with more retail purchases made locally and with income more equitably distributed. A similar study by MacCannell (1988) of comparable types of communities found that the concentration and industrialization of agriculture were associated with economic and community decline locally and regionally. Studies in Illinois (Gomez and Zhang 2000), Iowa (Durrenberger and Thu 1996), Michigan (Abeles-Allison and Conner 1990), and Wisconsin (Foltz et al. 2002) demonstrated decreased tax receipts and declining local purchases with larger operations. A Minnesota study (Chism and Levins 1994) found that the local spending decline was related to enlargement in scale of individual livestock operations rather than crop production. These findings consistently show that the social and economic well-being of local rural communities benefits from increasing the number of farmers, not simply increasing the volume of commodity produced (Osterberg and Wallinga 2004).

### Physical health

There have been more than 70 papers published on the adverse health effects of the confinement environment on swine producers by authors in the United States, Canada, most European countries, and Australia (Cormier et al. 1997; Donham 2000; Donham et al. 1977, 1982, 1986, 1990, 2002; Kirkhorn and Schenker 2002; Kline et al. 2004; Preller et al. 1995; Reynolds et al. 1996; Rylander et al. 1989; Schiffman et al. 1995; Schwartz et al. 1992; Thu et al. 1997; Wing and Wolf 2000). It is clear that at least 25% of confinement workers suffer from respiratory diseases including bronchitis, mucus membrane irritation, asthmalike syndrome, and acute respiratory distress syndrome. Recent findings substantiate anecdotal observations that a small proportion of workers experience acute respiratory symptoms early in their work history that may be sufficiently severe to cause immediate withdrawal from the work place (Dosman et. al. 2004). An additional acute respiratory condition, organic dust toxic syndrome, related to high concentrations of bioaerosols in livestock buildings occurs episodically in more than 30% of swine workers.

Environmental assessments of air quality inside livestock buildings reveal unhealthful concentrations of hydrogen sulfide, ammonia, inhalable particulate matter, and endotoxin (Iowa State University and University of Iowa 2002; Schenker et al. 1998). While there is less information on adverse effects among residents living in the vicinity of swine operations, that body of literature has been growing in recent years (Avery et al. 2004; Bullers 2005; Centers for Disease Control and Prevention 1998; Kilburn 1997; Merchant et al. 2005; Mirabelli et al. 2006a; Reynolds et al. 1997; Schiffman et al. 1995, 2000; Thu 2002; Thu et al. 1997; Wing and Wolf 2000).

Thu et al. (1997) documented excessive respiratory symptoms in neighbors of large-scale CAFOs, relative to comparison populations in low-density livestock-producing areas. The pattern of these symptoms was similar to those experienced by CAFO workers. Wing and Wolf (2000) and Bullers (2005) found similar differences in North Carolina. A case report associated with hydrogen sulfide exposure from a livestock processing facility in South Sioux City, Nebraska, revealed excessive diagnoses of respiratory and digestive disturbances in people living nearby (Campagna et al. 2004). Schiffman and colleagues reported that neighbors of confinement facilities experienced increased levels of mood disorders including anxiety, depression, and sleep disturbances attributable to exposures to malodorous compounds (Schiffman et al. 1995, 2000). Avery et al. (2004) found lower concentration and secretion of salivary immunoglobulin A among swine CAFO neighbors during times of moderate to high odor compared with times of low or no odor, suggesting a stress-mediated physiologic response to malodor (Shusterman 1992).

Community environmental air quality assessments have shown concentrations of hydrogen sulfide and ammonia that exceed U.S. Environmental Protection Agency (U.S. EPA) and Agency for Toxic Substances and Disease Registry recommendations (Reynolds et al. 1997). A recent study revealed that children living on farms raising swine have an increased risk for asthma, with increasing prevalence of asthma outcomes associated with the increased size of the swine operation (Merchant et. al. 2005). Children in North Carolina attending middle schools within 3 miles of one or more swine CAFOs and children attending schools where school staff report CAFO odors in school buildings were found to have a higher prevalence of wheezing compared with other middle school children (Mirabelli et al. 2006a, 2006b). It should be noted that these studies (although controlled) lack contemporaneous exposure assessment and health outcomes ascertainment. Additional research to include environmental exposure data related to biomarkers of response is needed.

### Mental health

Living in proximity to large-scale CAFOs has been linked to symptoms of impaired mental health, as assessed by epidemiologic measures. Greater self-reported depression and anxiety were found among North Carolina residents living near CAFOs (Bullers 2005; Schiffman et al. 1995). This finding was not corroborated in a small study by Thu et al. (1997) of depression among people living near to or far from CAFOs. However, it should be noted that the study of Thu et al. differed in that residents were not asked to report on their mental state during an actual odor episode as was the case in the study by Schiffman et al. (1995).

Greater CAFO-related posttraumatic stress disorder (PTSD) cognitions have been reported among Iowans living in an area of CAFO concentration compared with Iowans living in an area of a low concentration of livestock production (Hodne CJ, unpublished data). PTSD cognitions were consistent with interviewees’ multiple concerns about the decline in the quality of life and socioeconomic vitality caused by CAFOs, in areas of CAFO concentration with declining traditional family farm production.

### Social health

One of the most significant social impacts of CAFOs is the disruption of quality of life for neighboring residents. More than an unpleasant odor, the smell can have dramatic consequences for rural communities where lives are rooted in enjoying the outdoors (Thu 2002). The encroachment of a large-scale livestock facility near homes is significantly disruptive of rural living. The highly cherished values of freedom and independence associated with life oriented toward the outdoors gives way to feelings of violation and infringement. Social gatherings when family and friends come together are affected either in practice or through disruption of routines that normally provide a sense of belonging and identity—backyard barbecues and visits by friends and family. Homes are no longer an extension of or a means for enjoying the outdoors. Rather, homes become a barrier against the outdoors that must be escaped.

Studies evaluating the impacts of CAFOs on communities suggest that CAFOs generally attract controversy and often threaten community social capital (Kleiner AM, Rikoon JS, Seipel M, unpublished data; 2000; Ryan VD, Terry Al, Besser TL, unpublished data; Thu 1996). The rifts that develop among community members can be deep and long-standing (DeLind 1998). Wright et al. (2001), in an in-depth six-county study in southern Minnesota, identified three patterns that reflect the decline of social capital that resulted from the siting of CAFOs in all six rural communities they studied: *a*) widening gaps between CAFO and non-CAFO producers; *b*) harassment of vocal opponents of CAFOs; and *c*) perceptions by both CAFO supporters and CAFO opponents of hostility, neglect, or inattention by public institutions that resulted in perpetuation of an adversarial and inequitable community climate. Threats to CAFO neighbors have also been reported in North Carolina (Wing 2002). Clearly, community conflict often follows the siting of a CAFO in a community. What is not known is if community conflict resulting from the siting or presence of CAFOs has an impact on the ability of communities to act on other issues.

### Environmental injustice

Disproportionate location of CAFOs in areas populated by people of color or people with low incomes is a form of environmental injustice that can have negative impacts on community health (Wing et al. 2000). Several studies have shown that a disproportionate number of swine CAFOs are located in low-income and nonwhite areas (Ladd and Edwards 2002; Wilson et al. 2002; Wing et al. 2000) and near low-income and nonwhite schools (Mirabelli et al. 2006a, 2006b). These facilities and the hazardous agents associated with them are generally unwanted in local communities and are often thrust upon those sectors with the lowest levels of political influence. CAFOs are locally unwanted because of their emissions of malodor, nutrients, and toxicants that negatively affect community health and quality of life. Low-income communities and populations that experience institutional discrimination based on race have higher susceptibilities to CAFO impacts due to poor housing, low income, poor health status, and lack of access to medical care.

### Failure of the political process

In 2005 the U.S. Government Accountability Office issued a report on the effectiveness of U.S. EPA efforts in meeting its obligations to regulate concentrated animal feeding operations (U.S. Government Accountability Office 2005). The report identified two major flaws: *a*) allowing an estimated 60% of animal feeding operations in the United States to go unregulated, and *b*) lack of federal oversight of state governments to ensure they are adequately implementing required federal regulations for CAFOs. Additionally, many states have not taken a proactive stance to comply with the U.S. EPA regulations. Therefore, the concentration of livestock production, most noted by CAFO-style production, has continued to expand in most states. This has resulted in many rural communities and individuals taking action on their own, through local ordinances or litigation, as they have not been able to find access through usual governmental channels.

Several studies have found that property values decrease when CAFOs move into a community (Abeles-Allison and Conner 1990; Hamed et. al. 1999; Herriges et al. 2003; Palmquist et al. 1997). Neighbors of CAFOs are interested in preventing loss of property value, loss of their homes and land, forced changes in their life style, adverse changes in their communities, and threats to their health (Thu and Durrenberger 1998). The democratic process offers citizens access to lawmakers, to the courts, and to direct action to redress their grievances. However, the legislative process in many states has often been unresponsive to citizen wishes concerning CAFOs (Cantrell et al. 1996). For example, 13 states have enacted laws that inhibit citizens from speaking freely about agriculture if it is disparaging. A representative example can be seen in a South Dakota law that defines disparagement as

dissemination in any manner to the public of any information that the disseminator knows to be false and that states or implies that an agricultural food product is not safe for consumption by the public or that generally accepted agricultural and management practices make agricultural food products unsafe for consumption by the public. (South Dakota Codified Laws 2006)

All 50 states have some form of right-to-farm legislation. This legislation serves to protect farming operations from zoning laws or lawsuits that would overly restrict the ability of farmers to do business (Chapin et al. 1998; Hamilton 1998). Right-to-farm legislation varies from state to state but may include laws that prevent zoning from limiting farm practices that have substantial detrimental effects on neighbors, such as CAFO production. Right-to-farm laws may also include preemption of other actions of local government that normally could limit what businesses are allowed to do, known as home rule. For example, the Iowa Supreme Court has ruled that county governments cannot use home rule powers or protection of public health to promulgate laws that are more restrictive than state laws currently in force (Worth County Friends of Agriculture v. Worth County, Iowa, 2004). Although local governmental action has been limited by the bias toward agricultural producers, individual actions have not. Courts in several states have ruled that right-to-farm laws give only limited protection from nuisance action. The Iowa Supreme Court in June 2004 found that CAFO immunity provisions written in Iowa statutes were unconstitutional (Gacke v. Pork XTRA 2004). A district court in Illinois granted a temporary injunction stopping the construction of a nearby CAFO based on an anticipatory nuisance premise (Nickels et al. vs. Burnett 2002) that such a facility would constitute reasonable interference with neighbors’ quality of life.

Most states have enacted some forms of environmental laws aimed at protecting the environment from agricultural discharges or emissions. One form of these laws requires establishment of manure management plans. Typically, these laws call for certain sizes of operations to apply for permits. These permits may include the filing of a manure management plan, which calls for a plan for CAFO operators to manage their manure in a manner to prevent water and soil pollution. However, there is little if any performance inspection or enforcement of these plans (Jackson et al. 2000). Nonenforcement is primarily due to the lack of personnel and technical resources at state environmental agencies. For example, some states may have 2,000 or more such operations but not enough staff to efficiently process permit applications, much less get out into the field to inspect performance of these operations.

## Workshop Recommendations

### Priority research needs

#### Community health studies

Although sufficient research supports actions to protect rural residents from the negative impacts of CAFOs on community health, additional research could be conducted to further delineate mechanisms of effects and impacts on susceptible subgroups. These areas include psychophysiologic impacts of malodor; impacts of malodor on mental health and quality of life; and respiratory impacts of bioaerosol mixtures, especially among asthmatics, children, and the elderly. Wider and more effective application of community-based participatory research will be important to advance research in these areas.

#### Sustainability of livestock production

Federal funding for agricultural research should be reoriented to promote innovation in sustainable livestock production.

### Translation of science to policy

Requirements for issuing permits for CAFOs should include increased protections for health and the environment including the following:

- CAFOs should be sited and issued permits on the basis of total animal density allowed in a given watershed as determined by the carrying capacity.
- Environmental impact statements should be mandated for all new CAFOs. These should include environmental health, social justice, and socioeconomic issues.
- Decisions to issue permits for CAFOs should be considered in public meetings and decided at the local level.
- CAFOs should be regulated using standards applied to general industry based on the level of emissions and type of waste handling.
- Permits for manure storage basins should require bonding for performance and remediation.
- The current state of knowledge of community impacts of CAFOs warrants support for the American Public Health Association recommendation for a moratorium on all new CAFO construction.
